# ID 140 - Tomografia Computadorizada por Emissão de Pósitrons (PET-CT) para Estadiamento de Pacientes com Câncer de Esôfago: análises de custo-efetividade e de impacto orçamentário

**DOI:** 10.5327/2237-9622.2025.v34s1.140

**Published:** 2025-11-25

**Authors:** Mariana Millan Fachi, Tassiane Cristine Santos de Paula, Haliton Alves Oliveira, Rosa Camila Lucchetta, Layssa Andrade Oliveira

**Keywords:** tomografia por emissão de pósitrons combinada à tomografia computadorizada, câncer de esôfago, estadiamento do câncer, avaliação econômica em saúde

## Abstract

**Introdução:**

O câncer de esôfago, uma das principais causas de morte mundial, inclui o carcinoma de células escamosas e o adenocarcinoma. O estadiamento do câncer de esôfago é uma etapa crítica no planejamento do tratamento e na previsão do prognóstico dos pacientes, podendo ser realizada por exames de imagem como tomografia computadorizada (TC) e PET-CT (tomografia por emissão de pósitrons associada à tomografia computadorizada). No entanto, atualmente o Sistema Único de Saúde (SUS) dispõe apenas da TC para o estadiamento e, para alguns casos, o resultado da TC é inconclusivo. Dessa forma, esta análise avalia a custo-efetividade e o impacto orçamentário da PET-CT em relação à TC para detecção de metástase à distância em pacientes com câncer de esôfago sem tratamento prévio.

**Método:**

Foi conduzida uma avaliação econômica para estimar a razão de custo-efetividade incremental (RCEI) da PET-CT em relação à TC de tórax e/ou abdômen. Para análise de custo-efetividade utilizou-se um modelo híbrido, combinando uma árvore de decisão a um modelo de Markov, em que os desfechos avaliados foram QALY ganho e resultado verdadeiro ganho (verdadeiro positivo [VP]+ verdadeiro negativo [VN]), incluindo custo diretos médicos num horizonte temporal de 20 anos, sendo aplicada uma taxa de desconto de 5% para custos e desfechos. Além disso, foi realizada uma avaliação de impacto orçamentário (AIO), considerando diferentes cenários de adoção da PET-CT no horizonte temporal de cinco anos, e incluindo somente custos diretos das tecnologias comparadas.

**Resultados:**

A análise de custo-efetividade da PET-CT mostrou maior benefício clínico em termos de QALY ganho e resultado verdadeiro ganho comparado à TC. A RCEI foi de R$ 5.973 e R$ 11.280, respectivamente, estando o valor de RCEI por QALY ganho dentro do limiar estabelecido pela Conitec (R$ 40.000/QALY ganho). As análises probabilísticas confirmaram esse resultado, enquanto a análise determinística indicou que a sensibilidade e a especificidade do teste foram os parâmetros que mais impactaram na RCEI, sem, no entanto, exceder o limiar de disposição a pagar. Quanto ao impacto orçamentário, foi identificada uma população elegível variando de 17.725 a 18.112 por meio de abordagem epidemiológica, que para um market share com PET-CT variando de 20% a 100% entre o primeiro e o quinto ano, geraria um incremento de custo acumulado em cinco anos de R$ 113,7 milhões, enquanto em um cenário de 100% de incorporação já no primeiro ano, o impacto acumulado em cinco anos seria de R$ 188,8 milhões.

**Conclusão:**

Considerando o limiar de custo-efetividade recomendado pela Conitec, a PET-CT foi considerada custo-efetiva em termos de QALY ganho. Contudo, na análise de impacto orçamentário, considerando somente o custo de aquisição da tecnologia, observou-se que a incorporação de PET-CT para pacientes com resultados negativos para TC no SUS pode gerar um incremento de custo em cinco anos da análise. É importante considerar, porém, que esse incremento de custo considera apenas o impacto da adição da PET-CT ao estadiamento, mas para além da identificação dos casos inconclusivos, pode ter impacto significativo na conduta clínica, influenciando as decisões terapêuticas e alterando a intenção de tratamento, contribuindo assim para a redução de intervenções cirúrgicas desnecessárias.

